# Assessing progression of keratoconus: novel tomographic determinants

**DOI:** 10.1186/s40662-016-0038-6

**Published:** 2016-03-11

**Authors:** Joshua K. Duncan, Michael W. Belin, Mark Borgstrom

**Affiliations:** Department of Ophthalmology & Vision Science, University of Arizona, Tucson, AZ USA; University of Arizona, University Information Technology Services, Tucson, AZ USA

**Keywords:** Keratoconus, Tomography, Ectatic disease, Progression, Amsler-Krumeich, Scheimpflug, Collagen cross-linking

## Abstract

Several methods have been described in the literature to both evaluate and document progression in keratoconus, but there is no consistent or clear definition of ectasia progression. The authors describe how modern corneal tomography, including both anterior and posterior elevation and pachymetric data can be used to screen for ectatic progression, and how software programs such as the Enhanced Reference Surface and the Belin-Ambrosio Enhanced Ectasia Display (BAD) can be employed to detect earlier changes. Additionally, in order to describe specific quantitative values that can be used as progression determinants, the normal noise measurement of the three parameters (corneal thickness at the thinnest point, anterior and posterior radius of curvature (ARC, PRC) taken from the 3.0 mm optical zone centered on the thinnest point), was assessed. These values were obtained by imaging five normal patients using three different technicians on three separate days. The 95 % and 80 % one-sided confidence intervals for all three parameters were surprisingly small (7.88/4.03 μm for corneal thickness, 0.024/0.012 mm for ARC, and 0.083/0.042 mm for PRC), suggesting that they may perform well as progression determinants.

## Background

Keratoconus was first described in detail in 1854 as a chronic, non-inflammatory ectasia of the cornea. It is the most common primary ectasia, and is characterized by corneal steepening, visual distortion, apical corneal thinning, and central corneal scarring [1–3]. Corneal thinning typically occurs inferotemporal as well as central, although superior thinning has also been described [4]. Keratoconus usually becomes apparent during the second decade of the life, normally during puberty, and typically progresses until the fourth decade of life, when it usually stabilizes. The corneal thinning induces irregular astigmatism, myopia, and conical protrusion, leading to mild to marked impairment in the quality of vision, and often has a significant impact on patient’s quality of life [1]. Keratoconus is relatively uncommon with a reported annual incidence of 2 per 100,000 and prevalence of 54.5 per 100,000, though rates vary greatly in different geographic regions [5–7]. Keratoconus typically affects both eyes, although only one eye may be affected initially [8, 9]. The disease may be highly asymmetric [8, 9] and ocular symptoms and signs of keratoconus vary depending on disease severity. Early in the disease, and in subclinical keratoconus, there may be minimal or no symptoms, whereas in advanced disease there is significant distortion of vision accompanied by profound visual loss [10].

Several classification systems for keratoconus have been proposed in the literature [11–19]. The Amsler-Krumeich (AK) system is amongst the oldest and still the most widely used. In the AK system, the severity of keratoconus is graded from stage 1–4 using spectacle refraction, central keratometry, presence or absence of scarring, and central corneal thickness [20]. Others have used this system with various modification and additions in an attempt to better diagnosis or characterize the severity of disease [21, 22].

## Review

### Documenting ectatic progression

In addition to the various classification and grading systems described in the literature, having a standardized method to document ectatic progression is equally, if not more, important. The clinical decision to recommend treatments such as corneal crosslinking is based largely on documented progressive ectasia. According to Global Consensus on Keratoconus and Ectatic Diseases (2015), there is no consistent or clear definition of ectasia progression [23]. This panel defined progression by a consistent change in at least two of the following parameters: steepening of the anterior corneal surface, steepening of the posterior corneal surface, and thinning and/or thinning or changes in the pachymetric rate of change, nevertheless the panel also agreed that specific quantitative data to define progression is lacking [23].

Several methods have been described in the literature to both evaluate and document progression in keratoconus. Early and more recent systems utilized serial topographic analysis alone to attempt to document disease progression [24, 25], whereas a number of newly proposed systems use complex keratometric indices to describe progression [22, 26].

Kmax (maximum anterior sagittal curvature) is the most commonly used parameter to detect or document ectatic progression and is regularly used as an indicator for crosslinking’s efficacy [27–29]. Epstein et al. recommend the use of Kmax as a good single criterion to diagnose progression of keratoconus [30]. Kmax, however, has been acknowledged as a poor parameter for both progression and crosslinking efficacy [31–35]. Kmax represents the steepest anterior corneal curvature taken from a small area [30]. Kmax fails to reflect the degree of ectasia, ignores the contribution of the posterior cornea to progression and marked ectatic progression can occur with no change or even a reduction in Kmax [32–34].

Kanellopoulos et al. looked at seven anterior surface Pentacam-derived topometric indices, concluding that the index of surface variance (ISV) and the index of height decentration (IHD) may be the most sensitive and specific criteria in the diagnosis and progression of keratoconus [22]. Others have looked at visual acuity, manifest refraction, and central corneal thickness as measures to follow ectatic progression, but these have also been found to be unreliable, and do not correlate well with severity of keratoconus [35–37]. A number of other parameters or systems have been advocated to document progression [22, 25, 26, 34–40]. These include; observing for change on the posterior elevation maps, change in best corrected distance visual acuity, reduction in apical corneal thickness, or an increase in anterior corneal asymmetry. However, to the best of our knowledge, none of these have been validated in peer-reviewed literature as methods to monitor progression. Additionally, these methods suffer from either being limited only to the anterior cornea or representing a small portion of the cornea, which may not properly depict changes in the ectatic region. Visual acuity methods are very variable, as many practitioners have seen how unpredictable these subjective measurements can be in a keratoconic patient [36]. Corneal thickness measurements are typically altered (thinned) after crosslinking, thus limiting its value to document progression as well [41] (Table 1).

**Table 1:** Previously suggested parameters used to determine progression of ectatic disease

Suggested Parameter	Value Representing Progression	Validated
Spherical power, and higher order irregular astigmatism [32, 35]	Positive Rate of Change per Year	No
Spherical component, regular astigmatism, decentration component, and higher order irregularity [37]	Positive Rate of Change per Year	No
Kmax (steepest K) [27, 38]	≥ 1.00 D increase	No
Kmax – Kmin [38]	≥ 1.00 D increase	No
Kmean (average of Kmax and Kmin)	≥ 0.75 D increase	No
Pachymetry [38]	≥ 2 % decrease in central thickness	No
Back optic zone radius of the best fitting contact lens [27]	0.1 mm or more decrease	No
Increase in the central K power [25]	≥ 1.50 D increase from baseline	No
Manifest cylinder [38]	Increase of ≥ 1.00 D in 24 months	No
Manifest refraction spherical equivalent change (MRSE) [27, 38]	≥ 0.50 D	No
ISV [22]	Specific values for each KCN stage	No
IHA [22]	Specific values for each KCN stage	No

*ISV* = index of surface variance, *IHA =* index of height asymmetry, *KCN = *keratoconus

**Fig. 1:**
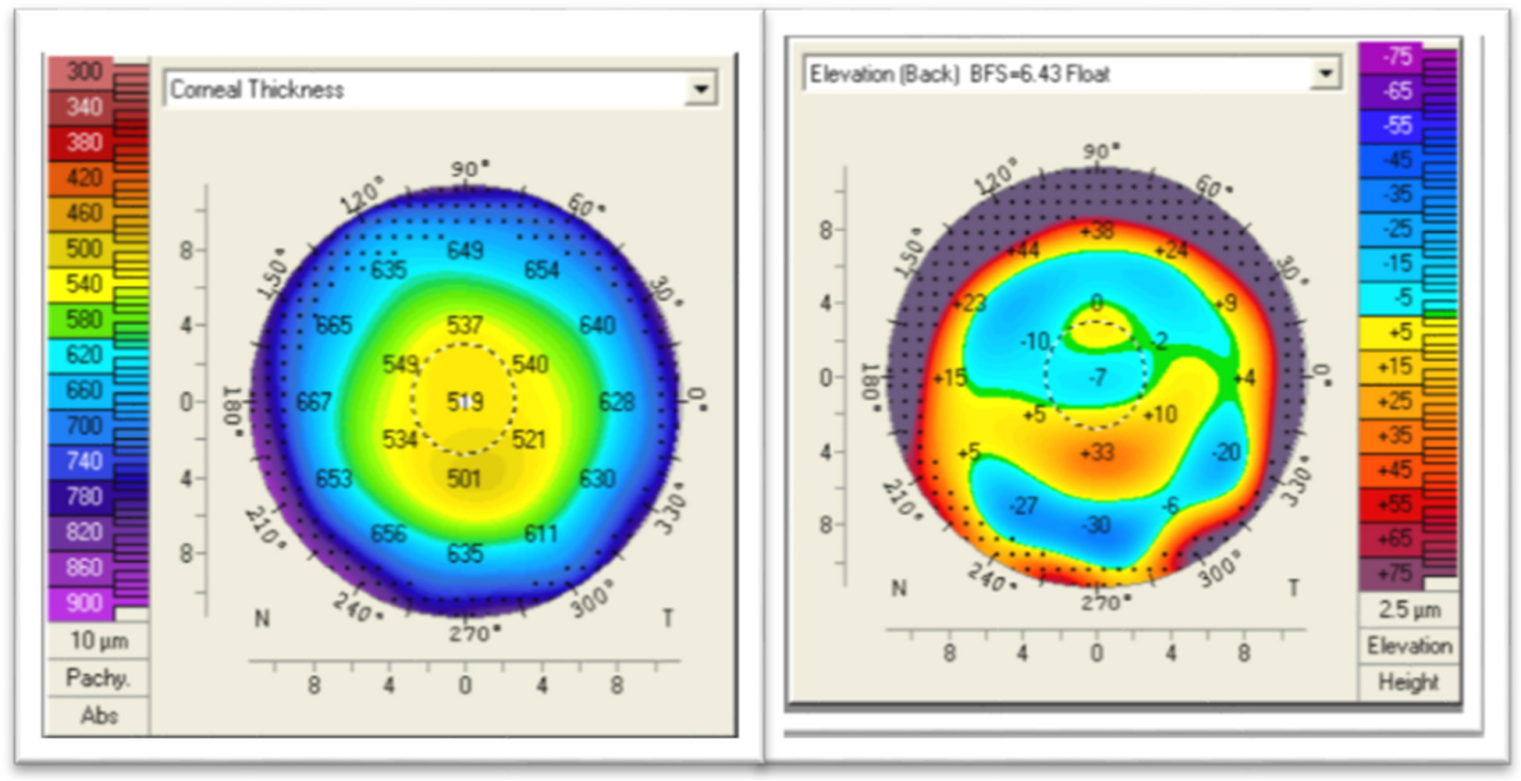
Corneal thickness map (*left*) and Posterior elevation (*right*). The corneal thickness map shows a thinnest point that is displaced inferiorly and the posterior elevation reveals a prominent posterior island in an eye that has a normal anterior surface (Oculus Pentacam)

It has been suggested that tomographic-derived pachymetry may be a more valuable method to document ectatic disease and follow progression [42]. Furthermore, changes in posterior corneal curvature [34], and corneal asymmetry have been shown to be additional methods of detecting early disease progression [22, 43, 44] (Fig. 1).

Other imaging techniques using Fourier series harmonic videokeratography and Fourier-Domain Optical Coherence Tomography (OCT) have been used to evaluate progression of keratoconus. Specifically, Oshika et al. looked at spherical power, regular astigmatism, decentration, and higher order irregular astigmatism as a means of quantifying advancement of ectasia [39]. OCT has been extensively utilized to evaluate total epithelial thickness, epithelial asymmetry, and biomechanical factors, which may be used to document progression of keratoconus [19]. The multitude of suggested progression parameters speaks to the need for a new or standardized method to document progression [23].

### Tomographic-based assessment of ectatic progression

**Fig. 2:**
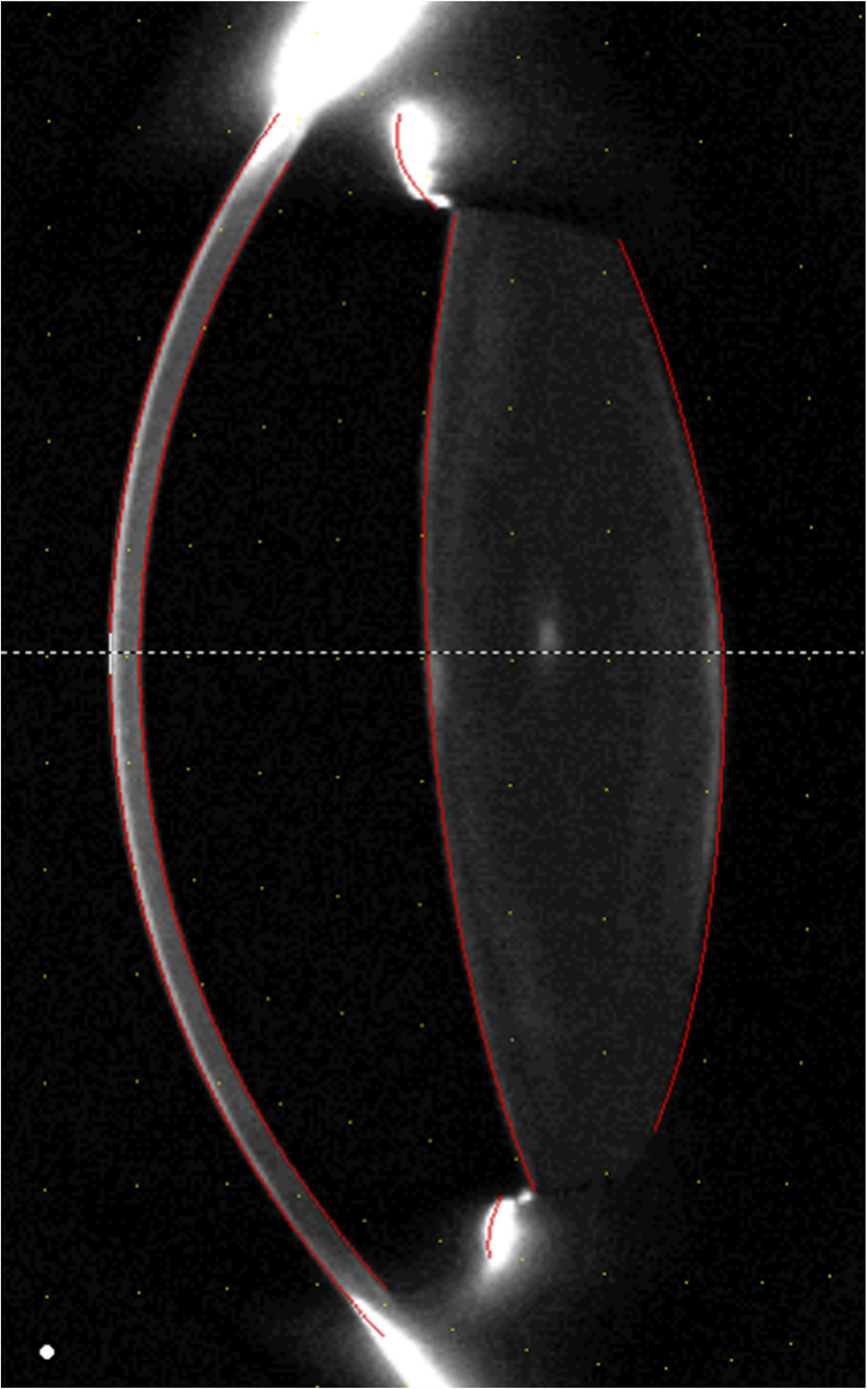
Scheimpflug optical cross section with edge detection turned on, showing the anterior corneal surface, posterior corneal surface, anterior and posterior lens surfaces identified (Oculus Pentacam)

Modern corneal tomography (as opposed to topography) allows for the measurement of the anterior and posterior corneal surfaces as well as the anterior lens (Fig. 2) [45].

**Fig. 3:**
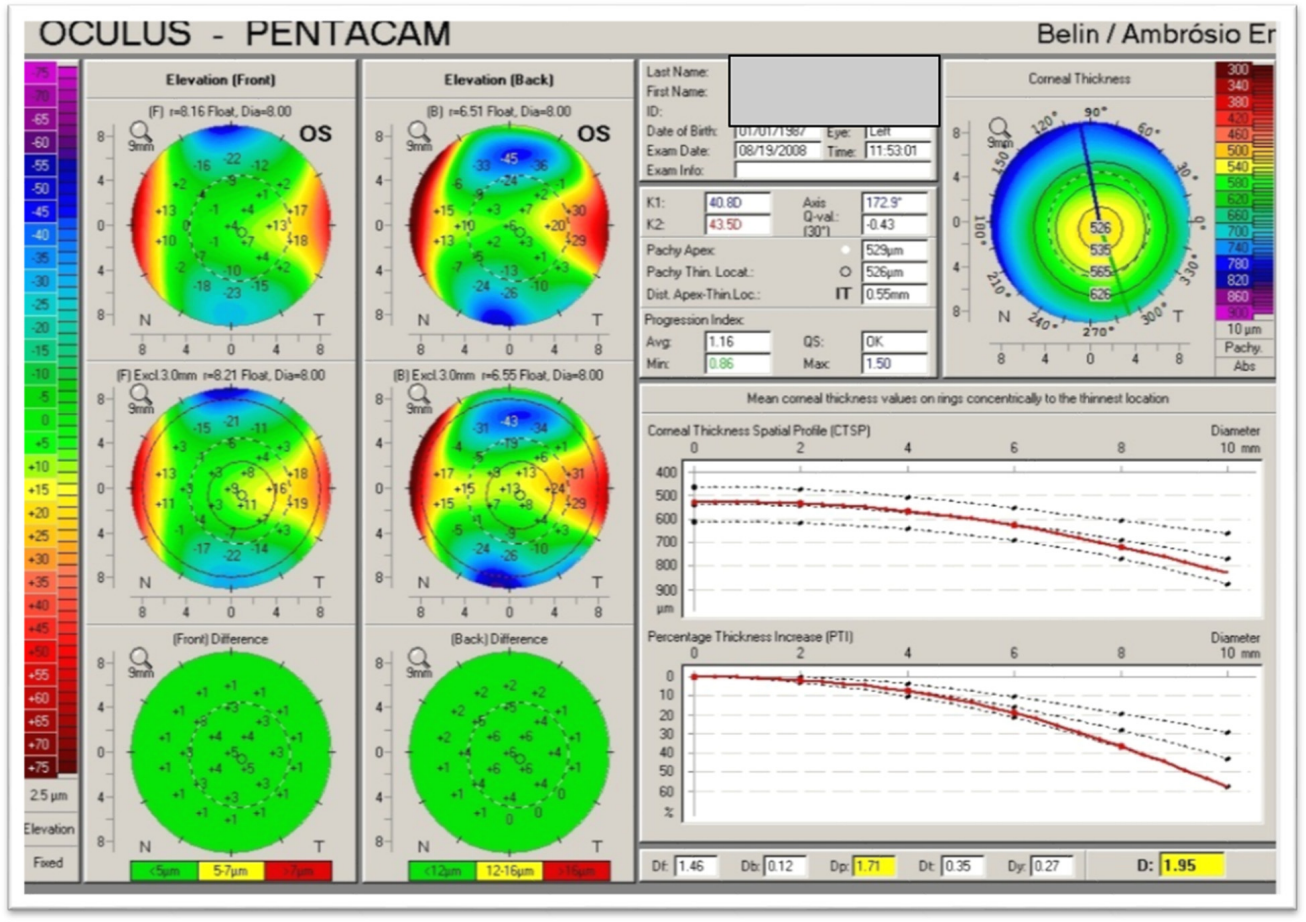
Contralateral eye in a patient with advanced keratoconus in the other eye. The only abnormality seen here (BAD display) is a mild abnormality in the pachymetric progression (Oculus Pentacam)

With this information, both corneal thickness and anterior chamber depth can be computed. Early ectatic change is typically seen on the posterior corneal surface prior to anterior changes (Fig. 1) [33]. Additionally, alterations in the corneal thickness, such as a more rapid change from the thinnest point to the periphery can be seen in early keratoconus even with normal anterior and posterior elevation maps (Fig. 3) [42].

The additional information available from anterior segment tomographic devices has led to the development of various refractive surgery screening programs. [14, 42, 46–48]. One such program is the Belin-Ambrosio Enhanced Ectrasia Display (BAD). The BAD display (available on the Pentacam, OCULUS GmbH, Wetzlar, Germany) utilizes both anterior and posterior elevation data and pachymetric data to screen for ectatic change [49, 50]. It displays the elevation data against the commonly used best-fit-sphere (BFS) taken from the central 8.0 mm zone, but also uses a newly developed reference surface called the “*Enhanced Reference Surface*.”

While the Best-Fit-Sphere (BFS) is both quantitatively and qualitatively useful, the clinician typically assumes that the reference surface closely approximates a “normal” cornea. This is actually not the case for ectatic corneas where the reference surface (typically a BFS taken from the central 8 mm zone) incorporates all data from the specified zone including normal and abnormal cornea [51]. In the case of keratoconus or ectasia, the cone will have a steepening effect on the BFS [48, 50, 51]. This steepened BFS will minimize the elevation difference between the apex of the cone and the BFS.

**Fig. 4:**
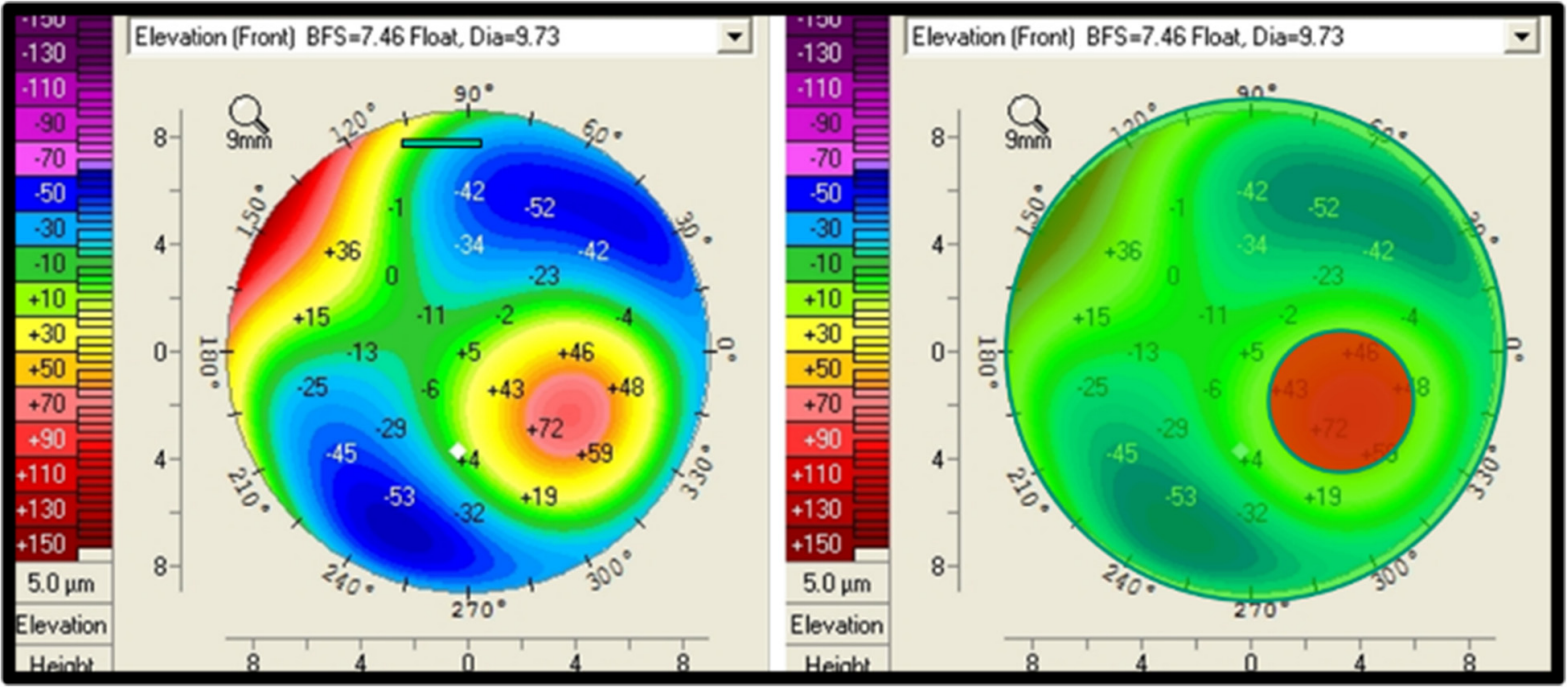
Anterior elevation map (left) showing a prominent paracentral positive island indicative of keratoconus. The map of the left highlights in red the 3.0 mm exclusion zone centered on the thinnest point that is removed from the calculation of the enhanced reference surface

The concept behind the “*Enhanced Reference Surface*” is to generate a reference surface that more closely resembles the patient’s own normal portion of the cornea as this will further magnify any existing pathology. To generate this new reference surface, a smaller diameter optical zone (exclusion zone) centered on the thinnest portion of the cornea is excluded from the 8.0 mm optical zone used for the standard BFS computation. The “*enhanced BFS*” is generated by utilizing all the valid elevation data from within the 8.0 mm central cornea, and outside the exclusion zone (Fig. 4).

**Fig. 5:**
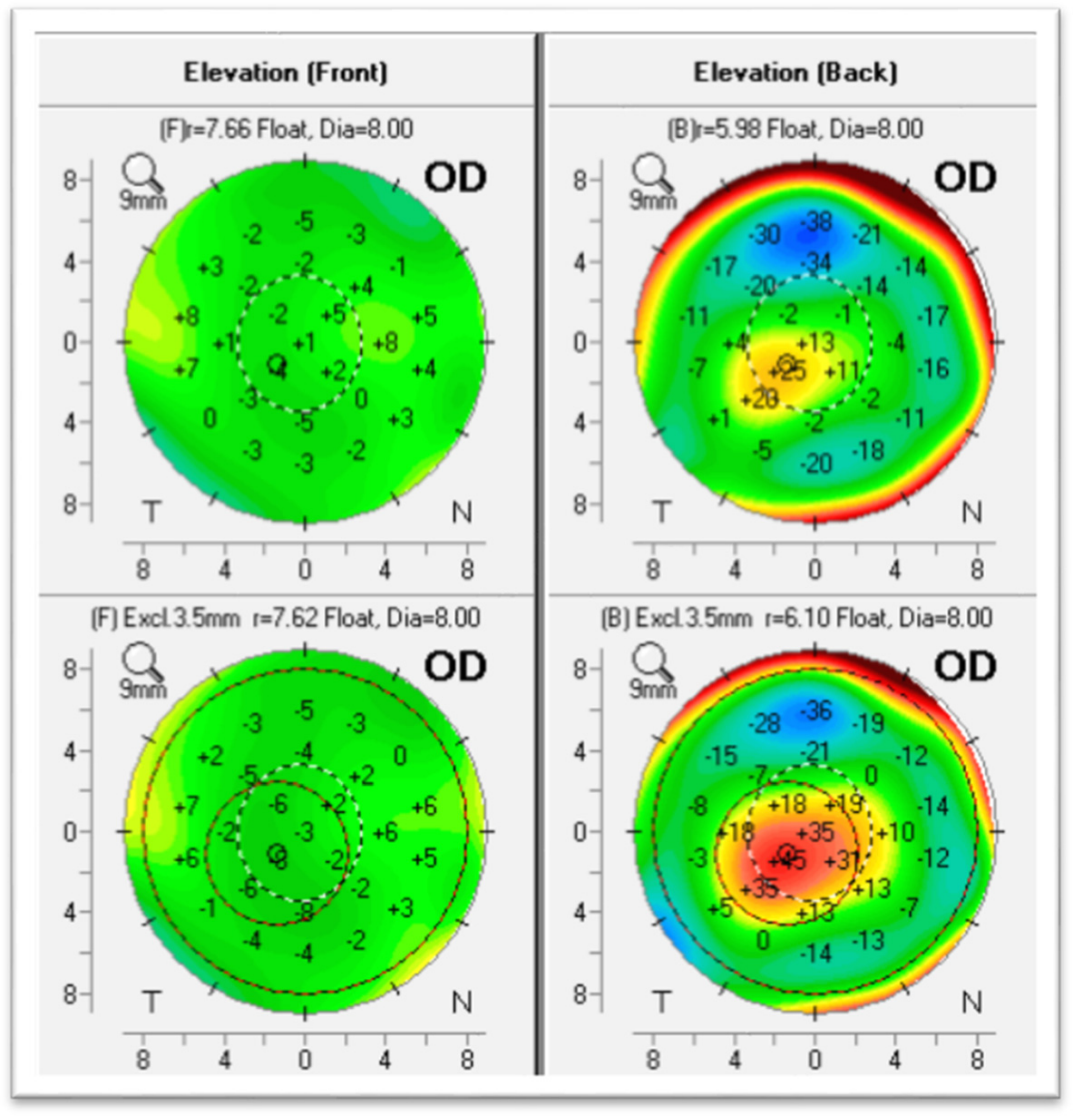
Anterior and Posterior elevation maps with the standard BFS (upper maps) and “*enhanced reference surface*” (lower maps). The standard anterior map (upper left) shows minimal changes against the enhanced reference surface (lower left) as the anterior surface is normal. The standard posterior elevation (upper right) shows an early positive island of elevation that is exaggerated using the enhanced reference surface (lower right) (Oculus Pentacam)

The exact size of the exclusion zone varies between 3.0 to 4.0 mm based on a proprietary algorithm, but is typically 3.0 mm for keratoconic corneas. The resulting new reference surface (“*Enhanced Reference Surface*) more closely approximates the more normal peripheral cornea and exaggerates any conical protrusion (Fig. 5).

The enhanced reference surface was not only qualitatively useful in visualizing subtle or early ectatic change, but the elevation difference between a standard BFS and the enhanced reference surface also proved to be highly significant quantitatively in separating normal eyes from those with ectatic change [50].

The choice of the exclusion zone centered on the thinnest point was multifactorial. The size of the exclusion zone had to be large enough to have more global representation than single parameters such as Kmax, but if the area was too large, then more “normal” cornea would be included; for displaced cones, far peripheral or extrapolated data would be incorporated. Extensive comparative testing resulted in the selection of a variable 3.0 to 4.0 mm exclusion zone [50, 51]. The enhanced reference surface works because the exclusion zone centered on the thinnest point incorporates the major ectatic region. Excluding this zone from the standard 8 mm BFS results in a reference surface that closely mimics the more normal portions of the cornea.

**Fig. 6:**
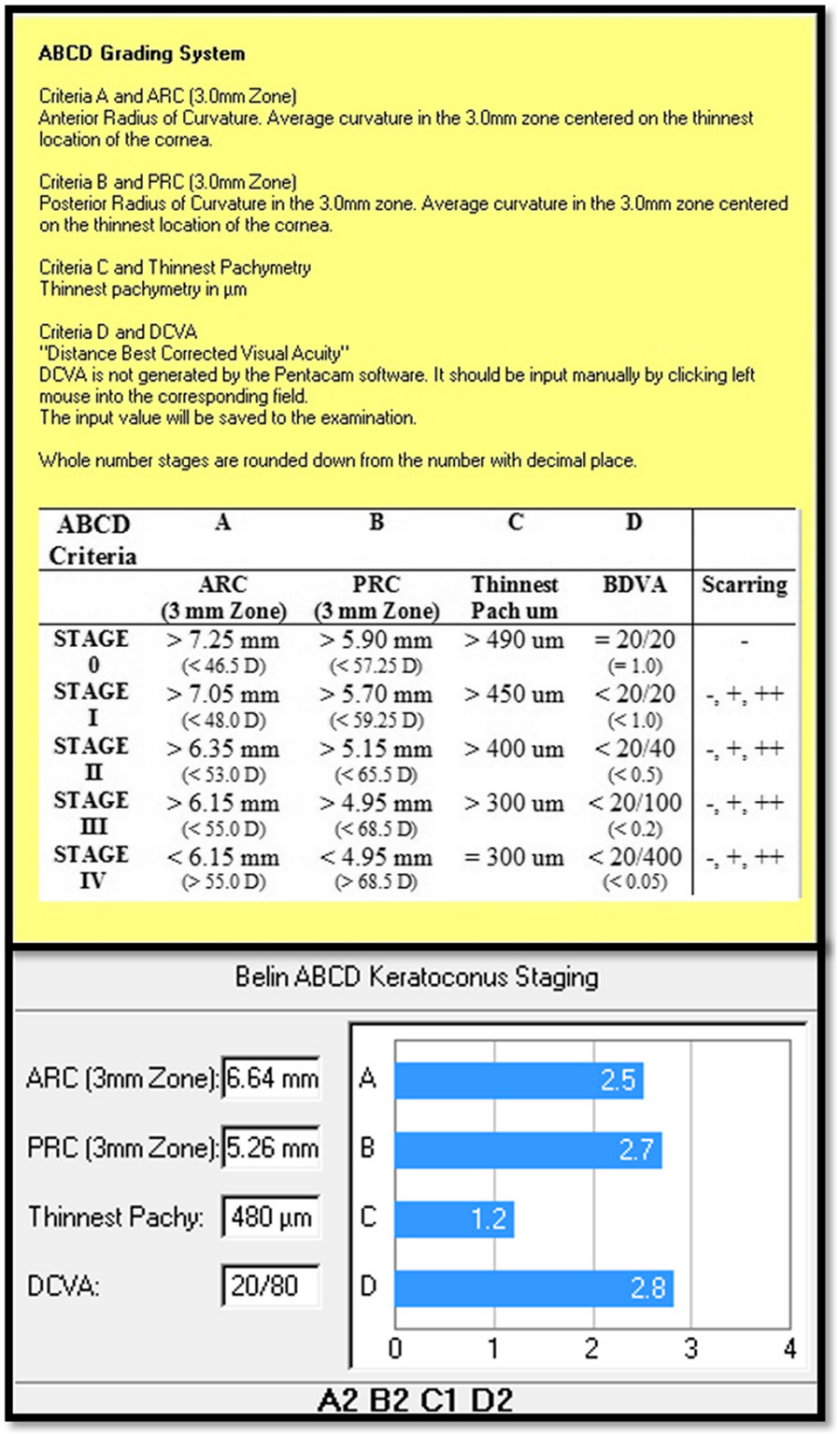
The ABCD Keratoconus Grading system currently available on the Topometric/Keratoconus Grading display on the OCULUS Pentacam

A similar concept has been used in a new keratoconus grading system [52, 53]. As opposed to excluding the 3.0 to 4.0 mm zone to normalize the reference surface, we employed the exclusion zone centered on the thinnest point as this area more globally represents the ectatic region than a single point parameter such as Kmax or maximal elevation. The newly described ABCD keratoconus grading system uses the anterior and posterior radius of curvature taken from the 3 mm zone centered on the thinnest point (“A” for anterior, “B” for back surface) and the corneal thickness at the thinnest point (“C” for corneal thickness) as well as best corrected distance visual acuity (“D” for distance visual acuity). This new classification/grading system has advantages over the older Amsler-Krumeich classification in that it recognizes the importance of the posterior corneal surface and each component (anterior, posterior, thickness, visual acuity) are individually graded. The “Belin ABCD” grading system has been incorporated in the OCULUS Pentacam software version 6.08r16 as part of the Topometric/Keratoconus Grading Display (Fig. 6).

**Fig. 7:**
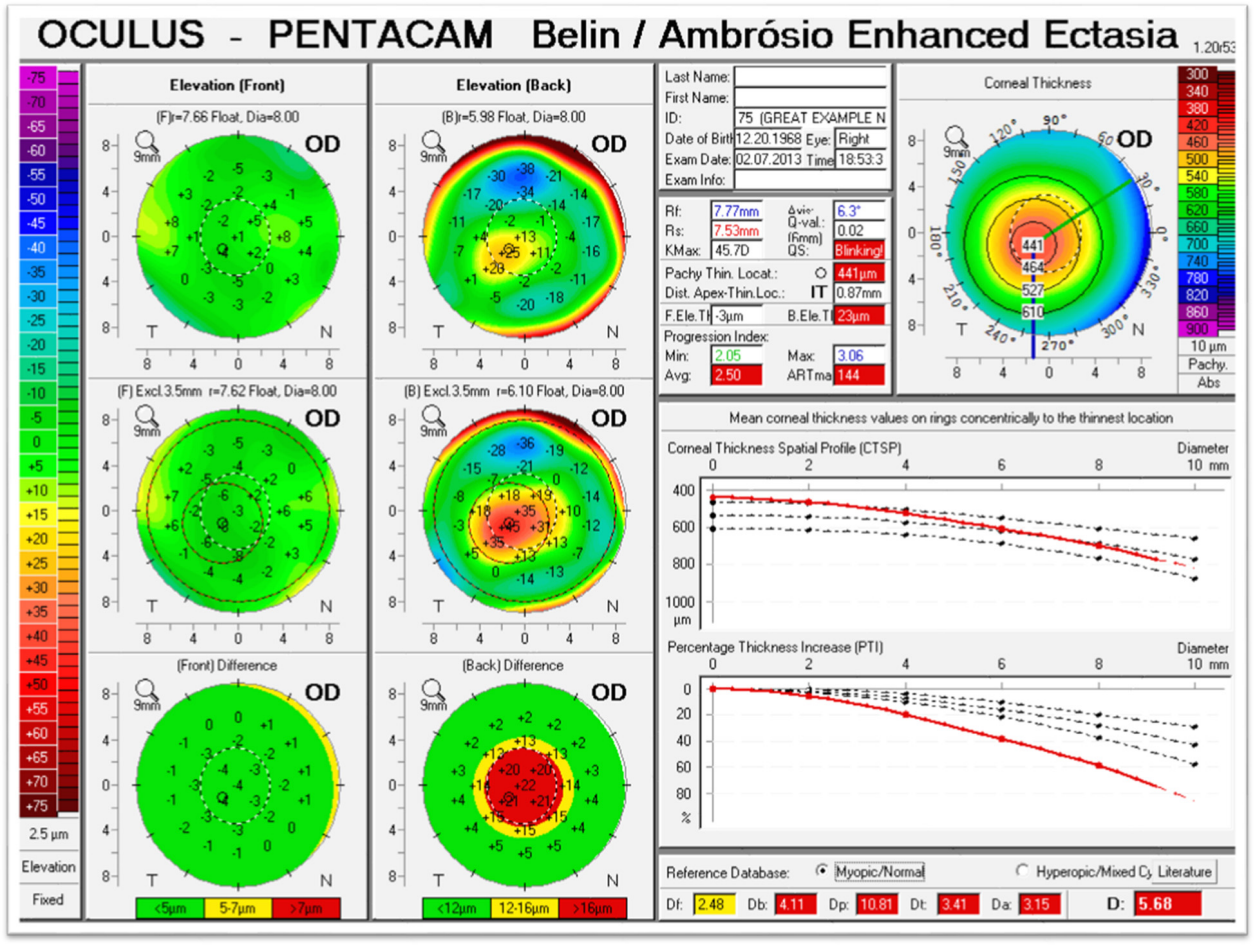
An example of subclinical keratoconus. The cornea is substantially thinned with a prominent posterior ectasia in spite of a normal anterior surface (BAD display, Oculus Pentacam)

Similarly, the determination of progression, or the lack of, is paramount to determine when and if to treat and to document treatment efficacy. As with the older grading systems, the problem with many of the commonly used progression parameters is that they were either limited to the anterior corneal surface (Kmax), or were measured on the corneal apex (Kmax, apical pachymetry) which often does not adequately reflect the cone. Changes in the cone may occur with little or any changes in the apical cornea. This would be particularly true for decentered cones. Additionally, changes on the posterior cornea may occur without concurrent anterior changes and they may be posterior progression in spite of a normal anterior surface (subclinical keratoconus) (Fig. 7). Progressive posterior ectasia will be accompanied by further corneal thinning, but this may not be detected only by taking measurements at the corneal apex.

Measuring corneal thickness change at the thinnest point should be a more sensitive indicator of progression than apical pachymetry. Changes to the anterior and posterior BFS taken from the 3.0 mm zone centered on the thinnest point should also be a more sensitive indicator of cone progression. The 3.0 mm zone was selected for the same reasons it was used in the ABCD grading system as this is the exclusion zone the BAD software chooses for most ectatic corneas. Because all three parameters are centered on the thinnest point (surrogate for center of the cone) and limited to the conical region, they should reflect change earlier than more global parameters (e.g. IHD, ISV) and/or parameters measured from the corneal apex. In order to utilize these parameters as indicators of progression, the normal measurement noise needs to be known. This allows us to separate measurement variance from true change. While numerous articles have been written on normal values generated by Scheimpflug imaging or OCT [48, 49, 54, 55], there are no available data on anterior and posterior curvature from the 3.0 mm zone centered on the thinnest point as these parameters have not been previously described.

To determine the measurement noise of the three parameters (corneal thickness at the thinnest point, and anterior and posterior radius of curvature (ARC, PRC) taken from the 3.0 mm optical zone centered on the thinnest point), five volunteer subjects were imaged, after obtaining informed consent, by three different technicians on three different days separated by 2 weeks (Pentacam HR, software version 6.08r13). Each technician imaged each patient three times for each time period for a total of 27 images per patient, 135 images total. Patients were removed from the instrument after each image. Each technician was instructed to acquire three images with an acceptable quality check (machine verification of an acceptable image). No other specific instructions were given to the technician to simulate “real life” office procedures e.g., variation in time of day. Specially designed software was used to extract ARC, PRC, and thinnest pachymetry (Table 2). The study protocol was approved by the University of Arizona (Tucson, Arizona) Institutional Review Board.

**Table 2:** Mean and Standard Deviation of each of the five subjects for thinnest pachymetry, ARC, and PRC

Patient	Minimal Pach (μm)	ARC from 3.0 mm zone (mm)	PRC from 3.0 mm zone (mm)
1	513.93 ± 6.49	7.35 ± 0.017	5.91 ± 0.033
2	521.81 ± 4.47	7.83 ± 0.016	6.40 ± 0.079
3	519.85 ± 3.02	7.43 ± 0.008	5.98 ± 0.033
4	491.37 ± 5.06	7.59 ± 0.011	6.21 ± 0.060
5	563.37 ± 4.23	7.83 ± 0.017	6.49 ± 0.027

*ARC =* anterior radius of curvature, *PRC =* posterior radius of curvature

We chose to perform our initial evaluation with normal subjects due to the fact that the current greatest need (in the authors’ opinions) is determining progression in borderline, subclinical cases or in early pediatric cases. Here, the normal patient variation is probably more applicable and more closely approximates very early disease than values determined from known cases of keratoconus. There are many surgeons who promote crosslinking in children at the first sign of ectatic change. Here, using parameters deduced from keratoconus patients would probably delay treatment. Additionally, while using cases of subclinical keratoconus would be germane, there still is no universal agreement on what constitutes subclinical disease, with many investigators still utilizing Amsler-Krumeich and relying on anterior surface topography [10, 23]. Future work, however, will evaluate patients with mild to moderate disease.

In order to determine the suitability of the above three parameters as potential progression determinants, both a pooled variance estimate and a one-sided confidence interval were computed using both SPSS version 23 (IBM Corp., Armonk, NY) and STATA 13 (StataCorp LP, College Station, TX). A one-sided confidence interval was chosen because progression is indicated by thinning and/or steepening of the anterior and/or posterior corneal surfaces. For each of these parameters (corneal thickness, ARC, PRC) a decrease would be indicative of progression. Both 95 % and 80 % confidence intervals were determined since the risk/benefit ratio for medical/surgical intervention would vary based on the age of the patient, family history, condition of the other eye, etc., (Table 3) and both the physician and patient’s decisions would differ greatly based on a multitude of factors.

**Table 3:** Standard deviation and 80 % and 95 % one-sided confidence intervals for corneal thickness, ARC and PRC for the pooled data

	Minimal Pach (μm)	ARC from 3.0 mm zone (mm)	PRC from 3.0 mm zone (mm)
Standard Dev	4.79	0.015	0.050
95 % one-tailed CI	7.88	0.024	0.083
80 % one-tailed CI	4.03	0.012	0.042

*ARC =* anterior radius of curvature, *PRC =* posterior radius of curvature, *CI =* confidence interval

## Conclusion

As earlier noted, according to Global Consensus on Keratoconus and Ectatic Diseases (2015), there is no consistent or clear definition of ectasia progression [23]. The panel defined progression by a consistent change in at least two of the following parameters: steepening of the anterior corneal surface, steepening of the posterior corneal surface, and thinning and/or thinning or changes in the pachymetric rate of change. The panel, however, acknowledged that specific quantitative data to define progression is lacking [23]. Our goal was to determine the quantitative values and to access their suitability as progression determinants. Both the 95 % and 80 % one-sided confidence intervals for all three parameters were surprisingly small (7.88/4.03 μm for corneal thickness, 0.024/0.012 mm for ARC, and 0.083/0.042 mm for PRC) suggesting that they may perform well as progression determinants. The limitation of the study is that the confidence intervals were determined on normal subjects and it is highly likely that measurement variability would be greater in ectatic corneas, though these values probably reflect early disease fairly well. The use of normal subjects was based on practical reasons since it would be difficult to have patients return on multiple days for measurements, though this is something we will pursue in the future. Finally, while minimal corneal thickness is readily available on all tomographic systems, ARC and PRC taken from the 3 mm zone centered on the thinnest point is a new parameter and currently only available on the OCULUS Pentacam, but would be simple to incorporate in any tomographic imaging system. The use of these parameters in addition to the ABCD grading system should offer an improved method of classifying and grading keratoconus and assist in documenting progression of disease.
